# Comparative Safety of GLP-1 Receptor Agonists Across Gastrointestinal, Renal and Pancreatic Systems

**DOI:** 10.3390/ph19010136

**Published:** 2026-01-13

**Authors:** Hala Shokr, Mohamed Mekkawy, Ali Hindi

**Affiliations:** 1 Division of Pharmacy and Optometry, School of Health Sciences, Faculty of Biology, Medicine and Health, The University of Manchester, Manchester M13 9PL, UK; 2 Clinical Pharmacy Department, Alexandria Police Hospital, Alexandria 21511, Egypt; ph.m.mekkawy@gmail.com

**Keywords:** obesity, weight loss injectables, semaglutide, liraglutide, tirzepatide

## Abstract

**Objectives:** to assess the safety profile of Gastrointestinal (GIT), renal, and pancreatic effects of GLP-1 and dual receptor agonists. **Methods:** Disproportionality analyses were performed on FDA Adverse Event Reporting System (FAERS) data following each drug’s approval for weight management. Signals were identified using Proportional Reporting Ratio (PRR) and Reporting Odds Ratio (ROR) with 95% confidence intervals (CIs). **Results:** Among GIT AEs, semaglutide (*n* = 12,321; 1.65%) showed the strongest signals (PRR 3.97, ROR 14.21), exceeding liraglutide (*n* = 5972, 0.45%, PRR 2.76, ROR 5.01) and tirzepatide (*n* = 4056, 3.48%, PRR 1.64, ROR 1.90). For renal and pancreatic outcomes, liraglutide demonstrated the highest overall signal (PRR 4.91, ROR 5.35), particularly for acute pancreatitis (PRR 18.9, ROR 19.4) and pancreatic carcinoma (PRR 18.6, ROR 19.5). Semaglutide showed stronger associations with diabetic ketoacidosis (DKA) (PRR 5.86, ROR 5.9) and acute kidney injury (AKI) (PRR 1.25, ROR 1.25). Tirzepatide demonstrated weaker or absent signals across most outcomes. **Conclusions:** This study revealed that semaglutide was most associated with GIT events, while liraglutide showed stronger renal and pancreatic signals. Novel associations with DKA and AKI were observed, warranting clinical vigilance. Findings should be cautiously interpreted given surveillance limitations, emphasising the need for large-scale real-world studies to confirm safety profiles.

## 1. Introduction

In the landscape of modern therapeutics, few drug classes have garnered as much clinical and commercial attention as glucagon-like peptide-1 (GLP-1) receptor agonists and the newer generation of dual incretin receptor agonists. Originally developed to treat type 2 diabetes mellitus (T2DM), these agents have rapidly evolved into cornerstone therapies, playing a pivotal role in addressing the global rise in obesity and related metabolic disorder [1]. Their widespread use stems from a growing body of evidence supporting their multifaceted clinical benefits: beyond improving glucose regulation, they promote sustained weight loss, enhance insulin sensitivity, reduce cardiovascular risk factors, and most notably in recent trials demonstrate nephroprotective effects that may alter the trajectory of diabetic kidney disease [2,3].

Drugs such as liraglutide, semaglutide, along with more recent dual agonists such as tirzepatide, which simultaneously target both GLP-1 and glucose-dependent insulinotropic polypeptide (GIP) receptors, are widely prescribed across a broad spectrum of patients, including individuals seeking pharmacological support for metabolic optimization [4]. These agents are lauded for their efficacy and durability of effect, yet their expanding role in clinical practice is not without risks [5].

Their use is associated with adverse effects, most notably involving the gastrointestinal (GIT) and the hepatobiliary, pancreatic, systems. While many of these side effects are self-limiting or manageable through dose titration, their persistence or severity can compromise adherence and quality of life [6]. This is particularly salient in non-diabetic populations using these drugs for obesity management, where the risk-benefit calculus may differ from that in the diabetic cohort.

A comprehensive understanding of the safety profile of GLP-1 and dual receptor agonists, particularly in routine clinical settings, requires consideration beyond the confines of randomized controlled trials. Although these trials are methodologically rigorous, they frequently exclude key demographic groups such as older adults, individuals with multiple comorbidities, and patients receiving complex medication regimens, thereby limiting the external validity of their findings. In contrast, post-marketing surveillance data offer a broader and more representative view of drug safety, capturing adverse event (AE) patterns across diverse and often underrepresented patient populations [7]. Additionally, given the increasing off-label use of these agents for weight management in otherwise healthy individuals, a deeper evaluation of their tolerability is warranted. Discrepancies between clinical trial data and post-marketing reports highlight the importance of integrating both sources to form a more complete and nuanced understanding of the safety profile.

The FDA AE Reporting System (FAERS) is a key resource in this context [8]. As a voluntary pharmacovigilance database, FAERS compiles reports of AE submitted by healthcare providers, patients, and manufacturers, offering a real-world perspective on the tolerability and risk profile of medications once they are in widespread clinical use. Leveraging this dataset allows clinicians and researchers to identify emerging safety signals, track patterns of AE, and refine clinical decision-making to ensure that the growing use of incretin-based therapies remains both effective and safe.

Previous studies using FAERS data have primarily focused on the safety profiles of semaglutide and liraglutide when they were first licensed for weight loss [9,10]. Whilst tirzepatide has been studied in isolation [11]. However, to date, no recent study has compared the safety profiles of all three agents. Furthermore, most studies have concentrated on GIT AE, with limited attention paid to renal and pancreatic outcomes despite close biological links and emerging evidence suggesting potential risks in these areas [12]. Comparing the safety profiles of these three drugs may provide better insights into personalised clinical management for obesity. The aim of this study is to explore the safety profile of GIT, renal, and pancreatic effects of GLP-1 and dual receptor agonists.

## 2. Results

During the FDA-approved timeframes for weight loss indications, January 2018 to June 2024 for semaglutide, January 2013 to June 2024 for liraglutide, and January 2023 to June 2024 for tirzepatide, a total of 744,788, 1,341,759, and 116,447 GIT AE reports, respectively, were identified in the FAERS database. Within these, the number of GIT AEs specifically attributed to each drug was: semaglutide (*n* = 12,321; 1.65%), liraglutide (*n* = 5972; 0.45%), and tirzepatide (*n* = 4056; 3.48%).

**Figure 1:**
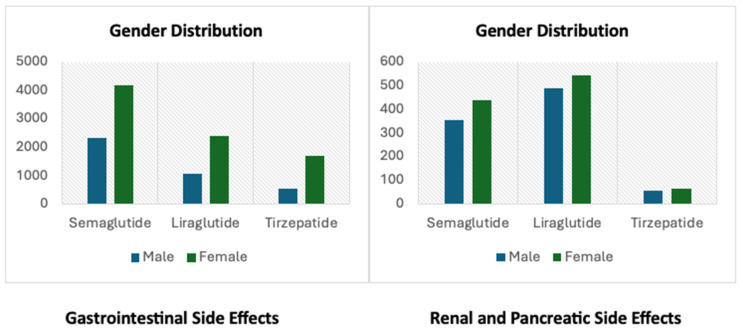
Gender Distribution Across Reported Side Effects.

**Figure 2:**
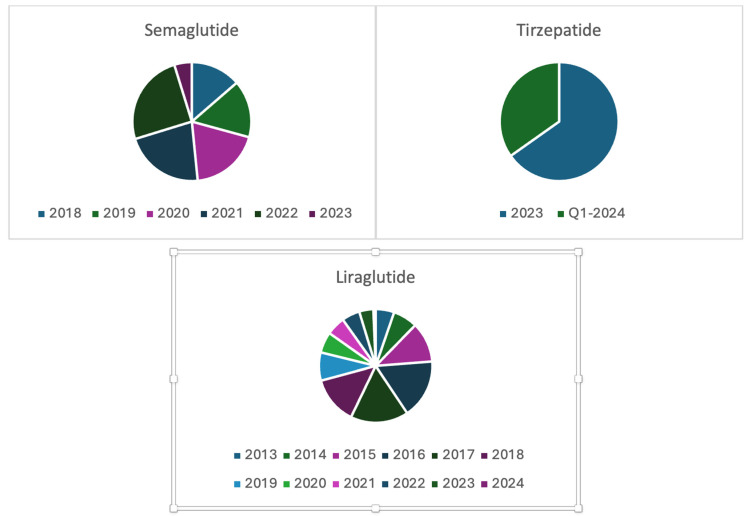
Reporting Years of Side Effects. For liraglutide, the year 2024 only had 23 reported cases. Hence, it is not visible on the graph.

The analysis of AEs for semaglutide, liraglutide and tirzepatide revealed distinct patterns in side effects. Across the three drugs, females predominated, as shown in Figure 1, with median ages of 63, 59, and 53 years, respectively, shown in Table 1. Age distributions showed a significant proportion 18–65 (semaglutide: 57.4%, liraglutide: 69.7%, tirzepatide: 74.1%,), while weight was almost equally distributed across these categories (<80 kg, 80–100 kg and >100 kg). Reports were mostly from the US for semaglutide (85.5%) and liraglutide (78.1%) and for tirzepatide (97.5%). Furthermore, the number of reported side effects for these drugs has increased since FDA approval for the management of obesity as shown in Figure 2.

### 2.1. Comparison of GIT Safety Profiles

Semaglutide demonstrated the strongest disproportionality signal for overall GIT adverse events, with a PRR of 3.97 (95% CI: 3.93–4.00) and an ROR of 14.21 (95% CI: 13.69–14.75). This signal was notably higher than that of liraglutide (PRR: 2.76; ROR: 5.01) and tirzepatide (PRR: 1.64; ROR: 1.90). The non-overlapping confidence intervals indicate a statistically significant difference in reporting patterns between the drugs. This trend was consistent across individual adverse events, including nausea, vomiting, diarrhoea, constipation, dehydration, and abdominal pain; see Table 2.

For abdominal discomfort and dizziness, semaglutide and liraglutide exhibited overlapping confidence intervals for both PRR (abdominal discomfort:2.89–3.47 and 2.65–3.32) (dizziness:1.66–1.9 and 1.2–1.45) and ROR (abdominal discomfort:2.95–3.55 and 2.69–3.4) and (dizziness:1.69–1.95 and 1.21–1.47), suggesting that the difference in signal strength between these two drugs may not be statistically significant. However, both drugs showed non-overlapping confidence intervals compared to tirzepatide, indicating stronger and statistically distinguishable signals relative to tirzepatide. For gastroparesis, semaglutide and liraglutide again showed overlapping confidence intervals for PRR (2.01–14.61 and 1.74–28.2) and ROR (1.69–1.95 and 1.74–28.2), implying no statistically significant difference between them for this AE and no reports linked to tirzepatide as shown in Table 2.

### 2.2. Comparison of Renal and Pancreatic Safety Profiles

Liraglutide demonstrated the strongest disproportionality signal for overall renal and pancreatic adverse events, with a PRR of 4.91 (95% CI: 4.64–5.20) and an ROR of 5.35 (95% CI: 5.02–5.70). These values were significantly higher than those for semaglutide (PRR: 2.76, 95% CI: 2.59–2.95; ROR: 2.86, 95% CI: 2.67–3.07) and tirzepatide (PRR: 0.57, 95% CI: 0.47–0.68; ROR: 0.56, 95% CI: 0.47–0.67), with non-overlapping confidence intervals, indicating a statistically significant difference in reporting patterns as shown in Table 3.

Semaglutide showed the strongest disproportionality signals for diabetic ketoacidosis (DKA) and acute kidney injury (AKI), with non-overlapping confidence intervals compared to liraglutide and tirzepatide, suggesting statistically significant differences for these specific events. For pancreatic carcinoma, liraglutide exhibited the highest disproportionality signal (PRR 18.6, ROR 19.48), followed by semaglutide (PRR 2.51, ROR 2.52) and then tirzepatide (PRR 1.4, ROR 1.4). Similarly, for acute pancreatitis, liraglutide again showed a stronger signal (PRR 18.93, ROR 19.35) than both semaglutide (PRR 6.26, ROR 6.3) and tirzepatide (PRR 1.2, ROR 1.2). In contrast, for nephrolithiasis, obstructive pancreatitis, bile duct stone, and cholecystectomy, semaglutide and liraglutide had overlapping confidence intervals for PRR and ROR, suggesting that the differences between them may not be statistically robust. However, both drugs showed non-overlapping confidence intervals compared to tirzepatide, indicating stronger signals relative to tirzepatide.

Additionally, all three drugs exhibited overlapping confidence intervals, implying that differences in disproportionality signals were not statistically meaningful for gallbladder disorder and appendicitis as shown in Table 3.

## 3. Discussion

The increasing clinical application of GLP-1 receptor agonists and dual incretin receptor agonists, such as semaglutide, liraglutide, and tirzepatide, has revolutionized the management of metabolic diseases. Their widespread use has extended beyond glycaemic control in type 2 diabetes to the management of obesity and cardiometabolic pathologies. However, this research showed that this therapeutic expansion has been accompanied by a notable burden of AE which has implications for patient adherence, quality of life, and clinical decision-making.

Demographically, the majority of AE across all drugs were reported by female patients and adults aged 18–65 years, a pattern that may partially reflect higher prescription rates in women for weight control purposes. The prominence of consumer-reported cases especially for tirzepatide (93.9%) suggests that patients are actively recognizing and reporting side effects, likely fuelled by increased public attention to these agents through media and direct-to-consumer advertising [13]. However, this also introduces potential biases, as consumer reports may lack medical verification and often underreport comorbidities or concurrent medications, limiting signal interpretation [8]. Importantly, a substantial proportion of AEs (approximately 30%) occurred in individuals weighing under 80 kg, pointing to increasing use among non-obese individuals, likely reflecting trends in non-medically supervised use of these drugs [13].

The temporal trends in AE reporting further highlight the influence of market dynamics. Reporting for semaglutide and tirzepatide surged following their respective approvals for weight management, supporting concerns raised in the literature about the safety of off-label or cosmetic use of metabolic drugs. For instance, semaglutide’s spike in 2023 coincides with its branding for obesity under the name Wegovy and subsequent social media-driven popularity [14,15]. This highlights a gap between regulatory-approved indications and real-world usage patterns, a concern that requires more rigorous post-marketing surveillance.

Despite their shared mechanisms as GLP-1 receptor agonists or dual agonists, differences in AE profiles have emerged. Comparative safety analyses consistently show that semaglutide is associated with the highest number of GIT AEs a pattern also reflected in previous FAERS data, where semaglutide demonstrated the highest risk of GIT side effects compared to liraglutide [16,17]. Additionally, our study found that tirzepatide exhibited a more favourable GIT tolerability profile. This may be attributed to its dual agonism of GLP-1 and GIP receptors, which could provide a more balanced metabolic effect and improved tolerability. Supporting this, a recent network meta-analysis found that the risk of GIT side effects with tirzepatide was not significantly greater than with other GLP-1 receptor agonists [18]. Notably, there have been no reports of gastroparesis associated with tirzepatide, further distinguishing its GIT safety profile from that of semaglutide and liraglutide.

Recent systematic reviews and meta-analyses have demonstrated that while GLP-1 receptor agonists (GLP-1 RAs) are associated with a statistically significant elevation in pancreatic enzyme levels such as amylase and lipase this biochemical finding has not translated into a clinically meaningful increase in the incidence of acute pancreatitis or pancreatic cancer when compared to placebo [19,20,21,22]. These findings suggest that enzyme elevations may reflect a pharmacologic effect rather than true pancreatic injury. Consistent with this, our study found no significant increase in pancreatic malignancy or pancreatitis across the GLP-1 RA class. However, when comparing individual agents, pancreatic AE including both acute pancreatitis and pancreatic carcinoma were reported more frequently with liraglutide. Nonetheless, causality cannot be inferred from spontaneous reporting data as high PRR/ROR values can be due to rare event reporting or notoriety bias, and further longitudinal studies are warranted to clarify these associations.

The mechanism underlying GLP-1-related pancreatitis is not fully understood but involves multiple factors: stimulation of pancreatic cell proliferation, altered enzyme secretion, slowed digestive motility, and potential effects on Reg protein expression, all of which may contribute to inflammation or duct obstruction [1]. In contrast, growing evidence suggests a more consistent association between GLP-1 RAs and gallbladder or biliary disease. Recent systematic reviews and meta-analysis reported a higher risk of these events particularly cholelithiasis among individuals receiving GLP-1 RAs at higher doses, for prolonged durations, and especially in the context of weight loss treatment [19,23]. Supporting these findings, our study observed a higher frequency of bile duct stones and cholecystectomy in patients receiving semaglutide and liraglutide compared to those on tirzepatide. These effects are thought to be class-related, likely mediated by GLP-1-induced alterations in gallbladder motility and biliary stasis.

Notably, our study identified a novel safety signal indicating a high reporting of nephrolithiasis and AKI associated with semaglutide. This potential renal risk has not been extensively characterized in the existing literature and may reflect a previously underappreciated aspect of semaglutide’s safety profile. Additionally, reports of DKA were more frequently linked to semaglutide compared to other agents in the class. Volume depletion from severe nausea, vomiting, and diarrhea has been highlighted as the main cause for GLP-1 induced AKI. Less commonly, direct drug effects like acute interstitial nephritis, an allergic-type kidney inflammation, or acute tubular necrosis can occur, even without GI symptoms, suggesting idiosyncratic reactions or off-target effects, with discontinuation often improving kidney function [24,25]. These findings highlight the importance of heightened clinical vigilance, particularly in individuals with predisposing factors for renal impairment or metabolic decompensation.

This study is the first to directly compare the safety profiles of these three drugs using real-world data from the FAERS database, one of the largest pharmacovigilance datasets. A key strength is its ability to assess post-marketing safety signals across a diverse, uncontrolled patient population over several years since drug approval, providing insights that may not emerge in clinical trials. Additionally, the large volume of reports allows detection of rare or unexpected adverse events.

Limitations include its retrospective design and reliance on spontaneously reported data from patients and healthcare professionals, which may be affected by underreporting, reporting bias, or incomplete information. While the observed safety signals suggest potential adverse effects, causality cannot be inferred from these data, and confounding factors such as dose escalation schemes, treatment duration, or patient comorbidity profiles may affect reporting patterns across drugs. Moreover, FAERS data are intended to indicate associations rather than establish causality. Lastly, time on the market may influence reporting patterns and impact comparative signal strength.

## 4. Materials and Methods

FAERS data were obtained from the publicly available FAERS Quarterly Data Extract Files [26]. Duplicate reports were identified using unique FAERS case identifiers, and follow-up reports were consolidated such that only the most recent version of each case was retained for analysis.

To assess changes in AE profiles following FDA approval for weight loss indications, data were analysed across the following timeframes: January 2018 to June 2024 for semaglutide, January 2013 to June 2024 for liraglutide, and January 2023 to June 2024 for tirzepatide. These periods correspond to each drug’s approval timeline for weight management.

The primary suspect drug was defined according to FAERS role code designation, and analyses were restricted to reports in which the study drug was listed as the primary suspect. Reports in which the drug was listed solely as a secondary or concomitant medication were not included.

Two established disproportionality analysis methods were employed: the Proportional Reporting Ratio (PRR) and the Reporting Odds Ratio (ROR). For each, 95% confidence intervals (CIs) were calculated to determine statistical significance. A signal for a potential adverse drug reaction (ADR) was considered present if the CI for either PRR or ROR excluded the null value of 1, indicating a significantly higher reporting frequency of that AE compared to other drugs.

To compare the strength of safety signals across the three medications, PRR and ROR values were evaluated side-by-side for each AE. When one drug exhibited a higher PRR or ROR and its confidence interval did not overlap with those of the other two medications, it was interpreted as having a statistically distinguishable and stronger signal for that specific AE.

## 5. Conclusions

This study is the first to compare the safety profiles of GLP-1 RAs, highlighting important differences between these agents. GIT adverse events were most frequently observed with semaglutide, whereas renal and pancreatic events appeared more commonly with liraglutide. Notably, new potential safety signals including DKA and AKI were identified, underscoring the need for heightened clinical vigilance. While these findings have direct implications for the monitoring and management of patients receiving GLP-1 RAs, they should be interpreted with caution given the limitations of post-marketing surveillance data and the inability to establish causality. Future studies, particularly large-scale real-world and mechanistic investigations, are essential to validate these associations, and guide evidence-based risk mitigation strategies in diverse patient populations.

## Figures and Tables

**Table 1:** Population Characteristics.

Age	Semaglutide	Liraglutide	Tirzepatide
Age (Median years)			
<18	17 (0.3%)	9 (0.3%)	0 (0%)
18–65	3030 (57.4%)	2078 (69.7%)	1416 (74.1%)
>65	2235 (42.3%)	896 (30%)	495 (25.9%)
Weight (Median Kg)			
<80	474 (28.1%)	330 (31.6%)	56 (29.5%)
80–100	567 (33.6%)	346 (33.1%)	66 (34.7%)
>100	646 (38.3%)	368 (35.3%)	68 (35.8%)
Reporting Countries			
US	5126 (85.5%)	2586 (78.1%)	2304 (97.5%)
Non-US	871 (14.5%)	724 (21.9%)	59 (2.5%)
Reporters			
Health care professional	1823 (27.8%)	938 (27.3%)	153 (6.1%)
Consumers	4737 (72.2%)	2496 (72.7%)	2355 (93.9%)

**Table 2:** Disproportionality Analysis of Gastrointestinal Adverse Events Associated with Semaglutide, Liraglutide, and Tirzepatide Using PRR and ROR Estimates.

	Semaglutide		Liraglutide		Tirzepatide		Significance
Side Effect	PRR (95% CI)	ROR (95% CI)	PRR (95% CI)	ROR (95% CI)	PRR (95% CI)	ROR (95% CI)	
GIT (all)	3.97 (3.93–4)	14.21 (13.69–14.75)	2.76 (2.71–2.8)	5.01 (4.82–5.21)	1.64 (1.59–1.68)	1.9 (1.83–1.97)	PRR: S > L > T ROR: S > L > T
Nausea	5.05 (4.9–5.2)	6.22 (6–6.46)	3.69 (3.54–3.85)	4.24 (4.03–4.46)	2.41 (2.29–2.54)	2.56 (2.42–2.72)	PRR: S > L > T ROR: S > L >T
Vomiting	5.63 (5.42–5.8)	6.45 (6.17–6.74)	3.73 (3.53–3.94)	4.05 (3.81–4.31)	1.61 (1.48–1.74)	1.63 (1.5–1.78)	PRR: S > L > T ROR: S > L > T
Diarrhoea	3.03 (2.9–3.16)	3.31 (3.16–3.48)	1.99 (1.87–2.13)	2.08 (1.94–2.23)	1.38 (1.28–1.49)	1.4 (1.3–1.51)	PRR: S > L > T ROR: S > L > T
Constipation	5.78 (5.46–6.1)	6.14 (5.78–6.53)	2.86 (2.59–3.16)	2.93 (2.64–3.25)	2.73 (2.47–3.02)	2.78 (2.51–3.08)	PRR:S > L= T ROR: S > L = T
Dehydration	4.52 (4.16–4.9)	4.64 (4.25–5.07)	2.84 (2.52–3.21)	2.89 (2.55–3.27)	1.51 (1.28–1.78)	1.52 (1.28–1.79)	PRR: S > L > T ROR: S > L > T
Abdominal pain	3.57 (3.4–3.75)	3.83 (3.63–4.04)	2.77 (2.59–2.96)	2.92 (2.72–3.14)	1.36 (1.24–1.49)	1.37 (1.24–1.51)	PRR: S > L > T ROR: S > L > T
Abdominal discomfort	3.17 (2.89–3.47)	3.23 (2.95–3.55)	2.97 (2.65–3.32)	3.02 (2.69–3.4)	1.89 (1.64–2.17)	1.9 (1.65–2.19)	PRR: S = L > T ROR: S = L > T
Stomach paralysis/ gastroparesis	5.42 (2.01–14.61)	5.42 (2.01–14.61)	7.01 (1.74–28.2)	7.01 (1.74–28.2)	No reports	No reports	PRR: S = L ROR:S = L
Dizziness	1.77 (1.66–1.9)	1.81 (1.69–1.95)	1.32 (1.2–1.45)	1.33 (1.21–1.47)	0.65 (0.57–0.73)	0.64 (0.56–0.73)	PRR: S = L > T ROR: S = L > T

Abbreviations: PRR: proportional reporting ratio; ROR: reporting odds ratio; S: Semaglutide; L: Liraglutide; T: Tirzepatide.

**Table 3:** Disproportionality Analysis of Renal and Pancreatic Adverse Events Associated with Semaglutide, Liraglutide, and Tirzepatide Using PRR and ROR Estimates.

	Semaglutide		Liraglutide		Tirzepatide		Comments
Side Effect	PRR (95% CI)	ROR (95% CI)	PRR (95% CI)	ROR (95% CI)	PRR (95% CI)	ROR (95% CI)	
Renal and pancreatic (all)	2.76 (2.59–2.95)	2.86 (2.67–3.07)	4.91 (4.64–5.2)	5.35 (5.02–5.7)	0.67 (0.57–0.79)	0.67 (0.56–0.79)	PRR: L > S > T ROR: L > S > T
Diabetic ketoacidosis	5.86 (4.97–6.91)	5.9 (5–6.97)	3.71 (2.91–4.73)	3.73 (2.92–4.76)	1.2 (0.78–1.85)	1.2 (0.78–1.85)	PRR: S > L > T ROR: S > L > T
Acute kidney injury	1.25 (1.08–1.44)	1.25 (1.08–1.45)	0.86 (0.71–1.05)	0.86 (0.7–1.05)	0.34 (0.25–0.47)	0.34 (0.25–0.47)	PRR: S > L > T ROR: S > L > T
Pancreatic carcinoma	2.51 (2.08–3.04)	2.52 (2.08–3.06)	18.6 (17.06–20.2)	19.48 (17.8–21.33)	1.4 (0.77–2.56)	1.4 (0.77–2.56)	PRR: L > S = T ROR L > S = T
Gallbladder disorder	5.07 (3.83–6.71)	5.08 (3.84–6.73)	4.26 (2.99–6.06)	4.27 (2.99–6.08)	2.08 (1.28–3.38)	2.08 (1.28–3.39)	PRR: L = S = T ROR L = S = T
Nephrolithiasis	1.83 (1.44–2.32)	1.83 (1.45–2.32)	1.9 (1.43–2.51)	1.9 (1.43–2.52)	0.69 (0.45–1.05)	0.69 (0.45–1.05)	PRR: S = L > T ROR: S = L > T
Obstructive pancreatitis	40.09 (27.8–57.65)	40.17 (27.92–57.8)	23.88 (13.08–43.6)	23.91 (13.09–43.67)	2.11 (0.51–8.7)	2.11 (0.51–8.7)	PRR: S = L > T ROR: S = L > T
Acute pancreatitis	6.26 (5.24–7.49)	6.3 (5.26–7.55)	18.93 (16.69–21.4)	19.35 (17.01–22.01)	1.2 (0.79–1.83)	1.2 (0.79–1.84)	PRR: L > S > T ROR: L > S > T
Appendicitis	3.67 (2.7–4.99)	3.67 (2.7–5)	1.73 (1–2.98)	1.73 (1–2.98)	0.86 (0.44–1.66)	0.86 (0.44–1.66)	PRR: L = S = T ROR L = S = T
Bile duct stone	18.47 (13.77–24.7)	18.52 (13.8–24.85)	9.4 (5.98–14.79)	9.42 (5.98–14.82)	0.61 (0.09–4.4)	0.61 (0.09–4.41)	PRR: S = L > T ROR: S = L > T
Cholecystectomy	5.37 (3.99–7.24)	5.38 (3.99–7.26)	6.07 (4.31–8.54)	6.08 (4.31–8.57)	0.12 (0.02–0.85)	0.12 (0.02–0.85)	PRR: S = L > T ROR: S = L > T

Abbreviations: PRR: proportional reporting ratio; ROR: reporting odds ratio; S: Semaglutide; L: Liraglutide; T: Tirzepatide.

## Data Availability

The original contributions presented in this study are included in the article. Further inquiries can be directed to the corresponding authors.

## References

[1] Liu Q.K. (2024). Mechanisms of Action and Therapeutic Applications of GLP-1 and Dual GIP/GLP-1 Receptor Agonists. Front. Endocrinol..

[2] The Evolving Story of Incretins (GIP and GLP-1) in Metabolic and Cardiovascular Disease: A Pathophysiological Update-Nauck-2021-Diabetes, Obesity and Metabolism-Wiley Online Library. https://dom-pubs.onlinelibrary.wiley.com/doi/full/10.1111/dom.14496.

[3] Bulum T. (2022). Nephroprotective Properties of the Glucose-Dependent Insulinotropic Polypeptide (GIP) and Glucagon-like Peptide-1 (GLP-1) Receptor Agonists. Biomedicines.

[4] Drucker D.J. (2024). Efficacy and Safety of GLP-1 Medicines for Type 2 Diabetes and Obesity. Diabetes Care.

[5] Pan X.-H., Tan B., Chin Y.H., Lee E.C.Z., Kong G., Chong B., Kueh M., Khoo C.M., Mehta A., Majety P. (2024). Efficacy and Safety of Tirzepatide, GLP-1 Receptor Agonists, and Other Weight Loss Drugs in Overweight and Obesity: A Network Meta-Analysis. Obesity.

[6] Ruder K. (2023). As Semaglutide’s Popularity Soars, Rare but Serious Adverse Effects Are Emerging. JAMA.

[7] Alomar M., Tawfiq A.M., Hassan N., Palaian S. (2020). Post Marketing Surveillance of Suspected Adverse Drug Reactions through Spontaneous Reporting: Current Status, Challenges and the Future. Ther. Adv. Drug Saf..

[8] U.S. Food and Drug Administration (2024). FDA Adverse Event Reporting System (FAERS) Public Dashboard. FDA. https://www.fda.gov/drugs/fdas-adverse-event-reporting-system-faers/fda-adverse-event-reporting-system-faers-public-dashboard.

[9] Shu Y., He X., Wu P., Liu Y., Ding Y., Zhang Q. (2022). Gastrointestinal Adverse Events Associated with Semaglutide: A Pharmacovigilance Study Based on FDA Adverse Event Reporting System. Front. Public Health.

[10] Zhou Y., Chen M., Liu L., Chen Z. (2022). Difference in Gastrointestinal Risk Associated with Use of GLP-1 Receptor Agonists: A Real-World Pharmacovigilance Study. Diabetes Metab. Syndr. Obes..

[11] Liu L. (2024). A Real-World Data Analysis of Tirzepatide in the FDA Adverse Event Reporting System (FAERS) Database. Front. Pharmacol..

[12] Ghusn W., Hurtado M.D. (2024). Glucagon-like Receptor-1 Agonists for Obesity: Weight Loss Outcomes, Tolerability, Side Effects, and Risks. Obes. Pillars.

[13] Basch C.H., Yousaf H., Hillyer G.C. (2025). Online Purchasing Options for GLP-1 Agonists: Accessibility, Marketing Practices, and Consumer Safety Concerns. J. Med. Surg. Public Health.

[14] Singh G., Krauthamer M., Bjalme-Evans M. (2022). Wegovy (Semaglutide): A New Weight Loss Drug for Chronic Weight Management. J. Investig. Med..

[15] Berning P., Adhikari R., Schroer A.E., Jelwan Y.A., Razavi A.C., Blaha M.J., Dzaye O. (2025). Longitudinal Analysis of Obesity Drug Use and Public Awareness. JAMA Netw. Open.

[16] Liu L., Chen J., Wang L., Chen C., Chen L. (2022). Association between Different GLP-1 Receptor Agonists and Gastrointestinal Adverse Reactions: A Real-World Disproportionality Study Based on FDA Adverse Event Reporting System Database. Front. Endocrinol..

[17] Zhou Q., Lei X., Fu S., Liu P., Long C., Wang Y., Li Z., Xie Q., Chen Q. (2023). Efficacy and Safety of Tirzepatide, Dual GLP-1/GIP Receptor Agonists, in the Management of Type 2 Diabetes: A Systematic Review and Meta-Analysis of Randomized Controlled Trials. Diabetol. Metab. Syndr..

[18] Pan H.-C., Chen J.-Y., Chen H.-Y., Yeh F.-Y., Sun C.-Y., Huang T.T.-M., Wu V.-C. (2024). GLP-1 Receptor Agonists’ Impact on Cardio-Renal Outcomes and Mortality in T2D with Acute Kidney Disease. Nat. Commun..

[19] Monami M., Dicembrini I., Nardini C., Fiordelli I., Mannucci E. (2014). Glucagon-like Peptide-1 Receptor Agonists and Pancreatitis: A Meta-Analysis of Randomized Clinical Trials. Diabetes Res. Clin. Pract..

[20] Monami M., Nreu B., Scatena A., Cresci B., Andreozzi F., Sesti G., Mannucci E. (2017). Safety Issues with Glucagon-like Peptide-1 Receptor Agonists (Pancreatitis, Pancreatic Cancer and Cholelithiasis): Data from Randomized Controlled Trials. Diabetes Obes. Metab..

[21] Wen J., Nadora D., Bernstein E., How-Volkman C., Truong A., Joy B., Kou M., Muttalib Z., Alam A., Frezza E. (2025). Evaluating the Rates of Pancreatitis and Pancreatic Cancer Among GLP-1 Receptor Agonists: A Systematic Review and Meta-Analysis of Randomised Controlled Trials. Endocrinol. Diabetes Metab..

[22] Guo H., Guo Q., Li Z., Wang Z. (2024). Association between Different GLP-1 Receptor Agonists and Acute Pancreatitis: Case Series and Real-World Pharmacovigilance Analysis. Front. Pharmacol..

[23] Annual General Meeting (AGM) GLP-1 RAs Associated with Increased Risk of Gallstones, Reflux, Meta-Analysis Finds. https://gastroenterology.acponline.org/archives/2025/06/27/1.htm.

[24] Tonneijck L., Smits M.M., Muskiet M.H.A., Hoekstra T., Kramer M.H.H., Danser A.H.J., Diamant M., Joles J.A., van Raalte D.H. (2016). Acute Renal Effects of the GLP-1 Receptor Agonist Exenatide in Overweight Type 2 Diabetes Patients: A Randomised, Double-Blind, Placebo-Controlled Trial. Diabetologia.

[25] Leehey D.J., Rahman M.A., Borys E., Picken M.M., Clise C.E. (2021). Acute Kidney Injury Associated with Semaglutide. Kidney Med..

[26] FAERS Quarterly Data Extract Files. https://fis.fda.gov/extensions/FPD-QDE-FAERS/FPD-QDE-FAERS.html.

